# Health behavior profiles and potential support needs among community residents with clustered cardiovascular risk factors: a latent profile and explainable machine learning analysis for community-based prevention

**DOI:** 10.1186/s12889-026-28868-y

**Published:** 2026-07-31

**Authors:** Kaihe Li, Xinyi Yan, Ying Zhou, Xuefen Lan, Xiaofen Li, Juanjuan Mai, Minqi Xia, Ruiming Chen

**Affiliations:** 1 School of Medicine, Lishui University, No. 1 Xueyuan Road, Lishui, Zhejiang 323000 China; 2 Department of Nursing, The First Affiliated Hospital of Lishui University, Lishui People’s Hospital, Lishui, 323000 Zhejiang China; 3 School of Medicine, Aksu Vocational and Technical College, Aksu Prefecture, Xinjiang, 843100 China

**Keywords:** Cardiovascular risk factors, Health behavior, Latent profile analysis, Explainable machine learning, Community-based prevention, Public health

## Abstract

**Background:**

Community residents with clustered cardiovascular risk factors may exhibit marked heterogeneity in health behavior patterns, posing challenges for community-based cardiovascular prevention and health behavior support. This study aimed to identify subgroups with different levels of health behavior and examine factors associated with subgroup membership using explainable machine learning.

**Methods:**

This secondary analysis used cross-sectional questionnaire data from 475 community residents with clustered cardiovascular risk factors. Health behaviors were assessed using the six dimensions of the Health-Promoting Lifestyle Profile II (HPLP-II). Latent profile analysis (LPA) was used to identify latent health behavior profiles that may reflect different behavioral support needs. The sample was split into training and internal validation sets at an 8:2 ratio. Variable selection was performed using Least Absolute Shrinkage and Selection Operator regression and Random Forest. Five classification models were developed and compared, and the model retained for exploratory analysis was subsequently interpreted using Shapley Additive Explanations (SHAP).

**Results:**

Four health behavior subgroups were identified: low health-promoting behavior profile (9.7%), low-to-moderate health-promoting behavior profile (45.5%), moderate-to-high health-promoting behavior profile (34.3%), and high health-promoting behavior profile (10.5%). In the internal validation set, the Random Forest (RF) model showed a comparatively balanced performance across multiple evaluation metrics, with an accuracy of 0.777, Kappa of 0.640, macro-averaged precision of 0.852, macro-averaged F1 score of 0.714, and area under the curve of 0.892; however, its minority-class identification requires further validation. SHAP analysis indicated that health behavior capability, social support, self-efficacy, and age were key contributors to subgroup classification.

**Conclusions:**

Health behaviors among community residents with clustered cardiovascular risk factors exhibit marked heterogeneity. These findings provide descriptive evidence of heterogeneity in health behaviors and may support stratified approaches to health behavior support and community-based cardiovascular prevention strategies.

**Supplementary Information:**

The online version contains supplementary material available at https://doi.org/10.1186/s12889-026-28868-y.

## Introduction

Cardiovascular disease (CVD) is one of the leading causes of death worldwide, and its occurrence and progression are closely associated with multiple risk factors, including hypertension, diabetes, dyslipidemia, obesity, smoking, and family history [1, 2]. According to the Global Burden of Disease study, the number of deaths from CVD worldwide increased from 12.4 million in 1990 to 19.8 million in 2022 [3], accounting for approximately 32% of global deaths and posing a substantial burden on global public health [2, 4]. Compared with individuals with a single risk factor, those with clustered cardiovascular risk factors have a greater risk of Major Adverse Cardiovascular Events (MACE) and present greater complexity for community-based prevention services [5, 6]. Community settings are critical for risk factor identification, health management, and long-term follow-up [7]. Identifying individuals with clustered cardiovascular risk factors in the community and promoting the formation and maintenance of healthy behaviors before adverse events occur are important tasks for community-based prevention services and chronic disease management.

Healthy behaviors refer to the various health-promoting practices individuals adopt to promote and maintain health and prevent disease [8]; improving these behaviors is a critical and modifiable intervention strategy for the prevention of cardiovascular disease [9]. Through the management of modifiable health behaviors—such as maintaining a balanced diet, engaging in regular physical activity, smoking cessation, alcohol moderation, and integrated control of blood lipids, glucose, and blood pressure [10, 11]—the incidence of CVD can be reduced, and the risk of MACE lowered [12]. Community health workers play a key role in cardiovascular disease prevention and control through health education, behavioral support, and follow-up management. Identifying the health behavior patterns of individuals with clustered cardiovascular risk factors and further clarifying the modifiable behavioral determinants of these patterns are therefore of considerable importance.

Health behavior gaps may vary across individuals. Previous studies have primarily employed a variable-centered approach, examining the relationship between a single health behavior or a total health behavior score and cardiovascular outcomes, with few investigations exploring the clustering characteristics and potential interactions of multiple health behaviors from a multidimensional perspective [13, 14]. Such approaches may obscure the heterogeneity of health behavior patterns within the population [15]. In the context of precision medicine, adopting a person-centered approach is necessary to identify potential subtypes of health behaviors among populations with clustered cardiovascular risk factors, thereby facilitating the development of tailored health education, behavioral support, and follow-up interventions.

Latent profile analysis (LPA), an unsupervised clustering technique, can identify subgroups of residents with similar behavioral characteristics across multiple dimensions of health behaviors [16]. Applying LPA to community residents with clustered cardiovascular risk factors allows for the elucidation of heterogeneity in the types and levels of health behaviors within these residents, thereby providing a foundation for community health workers to conduct stratified assessments and targeted interventions.

Identifying factors associated with membership in health behavior subgroups is a critical prerequisite for developing targeted intervention strategies. According to Social Cognitive Theory (SCT), health behaviors are shaped by the dynamic interaction of personal, behavioral, and environmental factors. Demographic characteristics, self-efficacy, health behavior capability, smoking and alcohol consumption, frequency of health checkups, and social support may all contribute to variations in health behavior patterns among populations with clustered cardiovascular risk factors [17–20]. Therefore, analyses of factors associated with different health behavior subgroups should consider not only individual-level characteristics but also behavioral and environmental determinants [21].

Although traditional regression models are commonly used to analyze associated factors, they typically require a prespecified model structure and may be limited in capturing complex and nonlinear relationships among variables [22]. Machine learning methods do not rely on a prespecified functional form and can identify complex patterns from multidimensional data, thereby improving the ability to detect factors associated with latent health behavior subgroup membership [23]. However, machine learning models often involve complex internal structures, which may make their results less intuitive and more difficult to interpret [24]. SHAP (Shapley Additive Explanations), which is based on the Shapley value principle from game theory, can quantify both the magnitude and direction of each variable’s contribution to model outcomes, thereby helping to identify key factors associated with membership in different health behavior subgroups [25, 26].

Among community residents with clustered cardiovascular risk factors, the main challenges in health behavior management may arise not only from insufficiency in a single behavior, but also from differences in health awareness, existing behavioral foundations, behavioral stability, and the capacity to maintain health behaviors. This heterogeneity makes it difficult for community teams to implement standardized health behavior management. In recent years, related health research has increasingly combined latent profile analysis with explainable machine learning to identify heterogeneous symptom or behavior patterns and to interpret key factors associated with subgroup membership [27], but evidence remains limited for applying this approach to community-based prevention services among residents with clustered cardiovascular risk factors. Translating the person-centered subgroup identification results obtained through LPA into interpretable classification evidence may help community health workers identify target groups with different health behavior support needs and support the preliminary development of stratified management plans. Therefore, guided by SCT, this study aimed to identify latent health behavior subgroups in this population and to use explainable machine learning to explore factors associated with subgroup membership. The findings may help identify subgroups that may differ in their potential behavioral support needs and provide preliminary evidence for stratified health behavior management in community public health practice.

## Methods

### Study design and participants

The sample size was calculated using the formula for cross-sectional surveys [28]: n = Zα/2² × P(1 − P) / δ². In this formula, n represents the minimum required sample size; Zα/2 represents the two-tailed critical value at the specified significance level under the standard normal distribution; P represents the expected proportion of the target population; and δ represents the allowable margin of error. With α = 0.05, Zα/2 = 1.96, and δ = 0.05, a conservative estimate was adopted by setting *P* = 0.50, at which P(1 − P) reaches its maximum value. The calculation yielded a minimum sample size of 385. After accounting for an anticipated 10% invalid questionnaire rate, the required sample size was 428. Ultimately, 475 valid questionnaires were included in this study, meeting the sample size requirement for a cross-sectional survey. The sample size was estimated using a general formula for cross-sectional surveys rather than a method specifically designed for LPA or machine learning classification. Although the current sample was sufficient to support exploratory subgroup identification and classification analyses, it may not have been large enough to ensure stable classification performance for the smaller latent profiles.

This study was a secondary analysis of data from a cross-sectional questionnaire survey and was reported in accordance with the STROBE guidelines. The survey was conducted from May to August 2024. Using convenience sampling, eligible participants were recruited from eight communities across four prefecture-level cities in Zhejiang Province, China: Lishui, Wenzhou, Hangzhou, and Shaoxing.

Operational definition of community residents with clustered cardiovascular risk factors: Based on national policies for CVD prevention and control, relevant studies on the clustering of cardiovascular risk factors, and established methods for CVD risk stratification, this study defined community residents with two or more traditional cardiovascular risk factors as having clustered cardiovascular risk factors [29–31]. Traditional risk factors included the following: family history of CVD or diabetes; hypertension or current use of antihypertensive medications; diabetes or current use of glucose-lowering medications; dyslipidemia, defined as serum total cholesterol > 5.2 mmol/L, triglycerides > 1.7 mmol/L, low-density lipoprotein cholesterol (LDL-C) ≥ 4.1 mmol/L, or high-density lipoprotein cholesterol (HDL-C) < 1.0 mmol/L, with meeting any one of these criteria considered positive for this item; obesity, defined as body mass index (BMI) ≥ 28 kg/m²; and smoking.

The inclusion criteria were as follows: (1) age ≥ 18 years; (2) being conscious and able to communicate effectively with the investigators; and (3) willingness to participate in the study. The exclusion criteria were as follows: (1) severe physical illness that prevented participation in the survey; and (2) severe mental illness or cognitive impairment that affected communication or questionnaire completion.

### Data collection

Data were collected using the Wenjuanxing platform, a commonly used online questionnaire survey platform in China, by five medical students who had received standardized training. All questions on the platform were set as mandatory to minimize missing data. With the assistance of community workers, the data collectors administered the survey through door-to-door visits and group-based survey sessions. Participants who were able to use electronic devices independently completed the electronic questionnaire by themselves, whereas those who had difficulty using electronic devices were assisted by trained interviewers using standardized instructions. After questionnaire submission, the researchers checked the completeness and logical consistency of the responses. A total of 500 questionnaires were received, of which 25 were excluded due to identical response patterns, yielding a valid response rate of 95.0% for the final analysis.

### Outcomes and measures

The Health-Promoting Lifestyle Profile II (HPLP-II) was used to assess participants’ health behaviors [32]. The scale was originally developed by Walker et al. [33], and its Chinese version has been used in the general Chinese population and among individuals with cardiovascular conditions [32, 34]. The HPLP-II consists of six dimensions: interpersonal relationships, nutrition, health responsibility, physical activity, stress management, and spiritual growth, with a total of 52 items. Each item is rated on a 4-point Likert scale ranging from 1 to 4. All items are positively scored, with higher scores indicating a higher level of health-promoting behaviors. The Cronbach’s α coefficients for the six dimensions range from 0.69 to 0.87, and the Cronbach’s α coefficient for the total scale is 0.90. In previous studies involving CVD populations, Cronbach’s α coefficients ranged from 0.82 to 0.90 [34], and a Cronbach’s α coefficient of 0.95 was reported in a previous study on health management among residents in Zhejiang Province [32]. In the present study, the Cronbach’s α coefficient for the total scale was 0.945, indicating good internal consistency.

### Candidate predictors of latent health behavior subgroup membership

Guided by SCT, this study selected relevant variables from three dimensions: individual-level, behavioral, and environmental factors. Individual-level factors included gender, age, occupation, marital status, educational level, monthly per capita household income, family history of disease, comorbidity of chronic diseases, self-efficacy, and health behavior capability. Behavioral factors included smoking status, alcohol consumption, and frequency of health checkups. Environmental factors included residential area, social support, and type of medical insurance.

Self-efficacy was measured using the General Self-Efficacy Scale (GSES), originally developed by Schwarzer and Jerusalem [35], adapted for Chinese populations by Zhang and Schwarzer [36], and further validated in Chinese samples by Wang et al. [37]. The scale consists of 10 positively scored items rated on a 4-point Likert scale, with total scores ranging from 10 to 40. Higher scores indicate greater general self-efficacy. In the present study, the Cronbach’s α coefficient was 0.868.

Health behavior capability was measured using the Chinese revised version of the Self-Rated Abilities for Health Practices Scale (SRAHP), originally developed by Becker et al. [38], translated into Chinese and validated by Huang and Chiou [39]. The scale consists of 26 items rated on a 5-point Likert scale, with total scores ranging from 0 to 104. Higher scores indicate stronger perceived ability to perform health-promoting behaviors. In the present study, the Cronbach’s α coefficient was 0.943.

Social support was measured using the Perceived Social Support Scale (PSSS), originally developed by Zimet et al. [40] and revised into Chinese by Jiang [41]. The scale consists of 12 items covering three dimensions: family support, friend support, and support from significant others. Each item is rated on a 7-point Likert scale, with total scores ranging from 12 to 84. Higher scores indicate greater perceived social support. In the present study, the Cronbach’s α coefficient was 0.943.

### Overall analytical procedure and descriptive statistics

All statistical analyses were performed using R version 4.5.3. LPA was conducted in the full analytical sample of 475 participants using the six HPLP-II dimension scores as manifest variables. The most likely profile assignments derived from the selected LPA solution were subsequently used as class labels in an exploratory machine learning classification analysis. Individual-level, behavioral, and environmental factors were included as candidate predictors for variable selection.

After the full-sample LPA was completed, the dataset was divided into a training set and an internal validation set at an 8:2 ratio using stratified sampling based on the LPA-derived profile assignments. The stratified split and all model training used fixed random seeds, and all hyperparameter tuning was conducted within the training set using 10-fold cross-validation. Variable selection, model training, and hyperparameter tuning were conducted within the training set. The tuned candidate models were subsequently compared in the internal validation set as an exploratory internal assessment. Because latent profile membership was derived from the full-sample LPA and the evaluation subset was used in the exploratory model comparison, this procedure was not considered an independent model validation. Baseline characteristics were described for the total sample, training set, and internal validation set. Categorical variables were presented as frequencies and percentages, and between-group comparisons were performed using the chi-square test. For 2 × 2 contingency tables, the chi-square test with continuity correction was used, whereas Fisher’s exact test was applied when the expected cell count was less than 5. Normally distributed continuous variables were expressed as mean ± standard deviation and compared using the independent samples t-test. Non-normally distributed continuous variables were expressed as median and interquartile range and compared using nonparametric tests. All tests were two-sided, and *P* < 0.05 was considered statistically significant.

Variable selection was performed using the training set. Least Absolute Shrinkage and Selection Operator (LASSO) regression and Random Forest (RF) were both used to screen candidate variables. In the LASSO regression, continuous variables were standardized using the default settings of the glmnet package. The optimal penalty parameter, λ, was determined through 10-fold cross-validation, and variables with non-zero regression coefficients were retained. In the RF model, class weights were assigned inversely proportional to class frequencies to reduce the model’s bias toward the majority classes and improve its ability to identify minority classes. Variables were further evaluated according to RF-based variable importance. The final set of variables included in the model was determined by integrating the LASSO regression results, RF variable importance rankings, the SCT framework, and clinical relevance.

### LPA

LPA was conducted using the full analytical sample before the data were divided for the exploratory machine learning analysis. LPA was conducted using tidyLPA version 2.0.2 with mclust version 6.1.2 as the estimation engine. Package-default random-start and convergence settings were used, and participants were assigned to the profile with the highest posterior probability. Using the scores of the six dimensions of the HPLP-II as manifest variables, latent profile analysis was conducted to identify distinct latent health behavior subgroups. Maximum likelihood estimation was used for model estimation. Model fit was evaluated using the Akaike Information Criterion (AIC), Bayesian Information Criterion (BIC), sample-size-adjusted Bayesian Information Criterion (SABIC), entropy, the Lo–Mendell–Rubin adjusted likelihood ratio test (LMR), and the bootstrapped likelihood ratio test (BLRT). The LMR and BLRT were used to compare the k-profile model with the k − 1-profile model; *P* < 0.05 indicated that adding one profile significantly improved model fit. Lower AIC, BIC, and SABIC values indicated better model fit. Entropy values range from 0 to 1, with values ≥ 0.80 indicating clearer classification and less overlap between profiles. The optimal profile solution was determined by considering model fit indices, the sample proportion of each profile, and practical interpretability.

### Machine learning model development and interpretation

LASSO regression, RF, support vector machine (SVM), extreme gradient boosting (XGBoost), and multilayer perceptron (MLP) neural network models were developed to classify latent health behavior subgroup membership derived from the full-sample LPA among community residents with clustered cardiovascular risk factors. Variable selection, model training, and hyperparameter tuning were conducted exclusively within the training set, during which class imbalance was addressed by assigning class weights inversely proportional to class frequencies.

For LASSO regression, 10-fold cross-validation was used to determine the optimal penalty parameter, λ. For RF, SVM, XGBoost, and MLP models, 10-fold cross-validation was conducted within the training set for model training and hyperparameter tuning. The tuned models were subsequently evaluated in the internal validation set. Model performance was comprehensively evaluated using accuracy, Kappa, precision, recall, F1 score, and multiclass area under the receiver operating characteristic curve (multiclass AUC). Precision, recall, and F1 score were calculated as macro-averages and weighted averages across the four subgroups, whereas multiclass AUC was calculated using the Hand and Till method.

Following the exploratory comparison of the tuned candidate models, multiclass AUCs were compared between the RF model and each alternative model using paired bootstrap resampling in the internal validation set, with Holm adjustment for multiple comparisons, as a supplementary post hoc analysis. SHAP analysis was applied to the model retained for downstream interpretation. Class-specific SHAP values were calculated to quantify the magnitude and direction of each variable’s contribution to the predicted probability of membership in each latent subgroup. Larger absolute SHAP values indicated greater contributions of the corresponding variables to the model’s classification output. Detailed variable coding, hyperparameter settings, and additional reproducibility information are provided in Additional Tables S2, S3, and S6.

### Ethical considerations

This study was a secondary analysis of data from a cross-sectional questionnaire survey conducted from May to August 2024. The original survey was conducted in accordance with the Declaration of Helsinki. Before data collection, participants were informed of the study purpose, the voluntary nature of participation, and the anonymous use of their responses, and informed consent was obtained from all participants. The Lishui University Ethics Committee approved the analysis and publication of the anonymized data.

## Results

### Latent health behavior profiles

Table 1 presents the fit indices for the one- to six-profile models, using the scores of the six dimensions of the HPLP-II as manifest variables. The results showed that AIC, BIC, and SABIC generally decreased as the number of profiles increased, and both the LMR and BLRT were statistically significant. Although the five-profile model had the highest entropy value of 0.873, the smallest profile accounted for only 2.9% of the sample, suggesting a potential risk of overextraction. The six-profile model also included small profiles, which may have limited its stability. The four-profile model had an entropy value of 0.868, with each profile accounting for more than 5% of the total sample, and the average posterior probabilities ranged from 0.918 to 0.954 (Additional Table S1). Considering the model fit indices, classification quality, profile size, and interpretability in public health practice, the four-profile model was ultimately selected as the optimal profile solution.

**Table 1:** Fit indices of the LPA models

Profile	AIC	BIC	SABIC	Entropy	LMR	BLRT	Proportion
C = 1	8105.943	8155.903	8117.817	1.000	-	-	1.000
C = 2	7073.716	7152.819	7092.516	0.870	*P* < 0.001	*P* < 0.010	0.693/0.307
C = 3	6664.922	6773.168	6690.648	0.849	*P* < 0.001	*P* < 0.010	0.429/0.432/0.139
C = 4	6480.677	6618.066	6513.329	0.868	*P* < 0.001	*P* < 0.010	0.097/0.455/0.343/0.105
C = 5	6331.232	6497.764	6370.810	0.873	*P* < 0.001	*P* < 0.010	0.095/0.320/0.158/0.398/0.029
C = 6	6248.085	6443.760	6294.589	0.865	*P* < 0.001	*P* < 0.010	0.095/0.259/0.154/0.385/0.078/0.029

Entropy indicates classification accuracy, with values closer to 1 representing clearer profile separation

*AIC* Akaike information criterion, *BIC* Bayesian information criterion, *SABIC* sample-size-adjusted Bayesian information criterion, *LMR* Lo–Mendell–Rubin adjusted likelihood ratio test, *BLRT* bootstrap likelihood ratio test

**Fig. 1:**
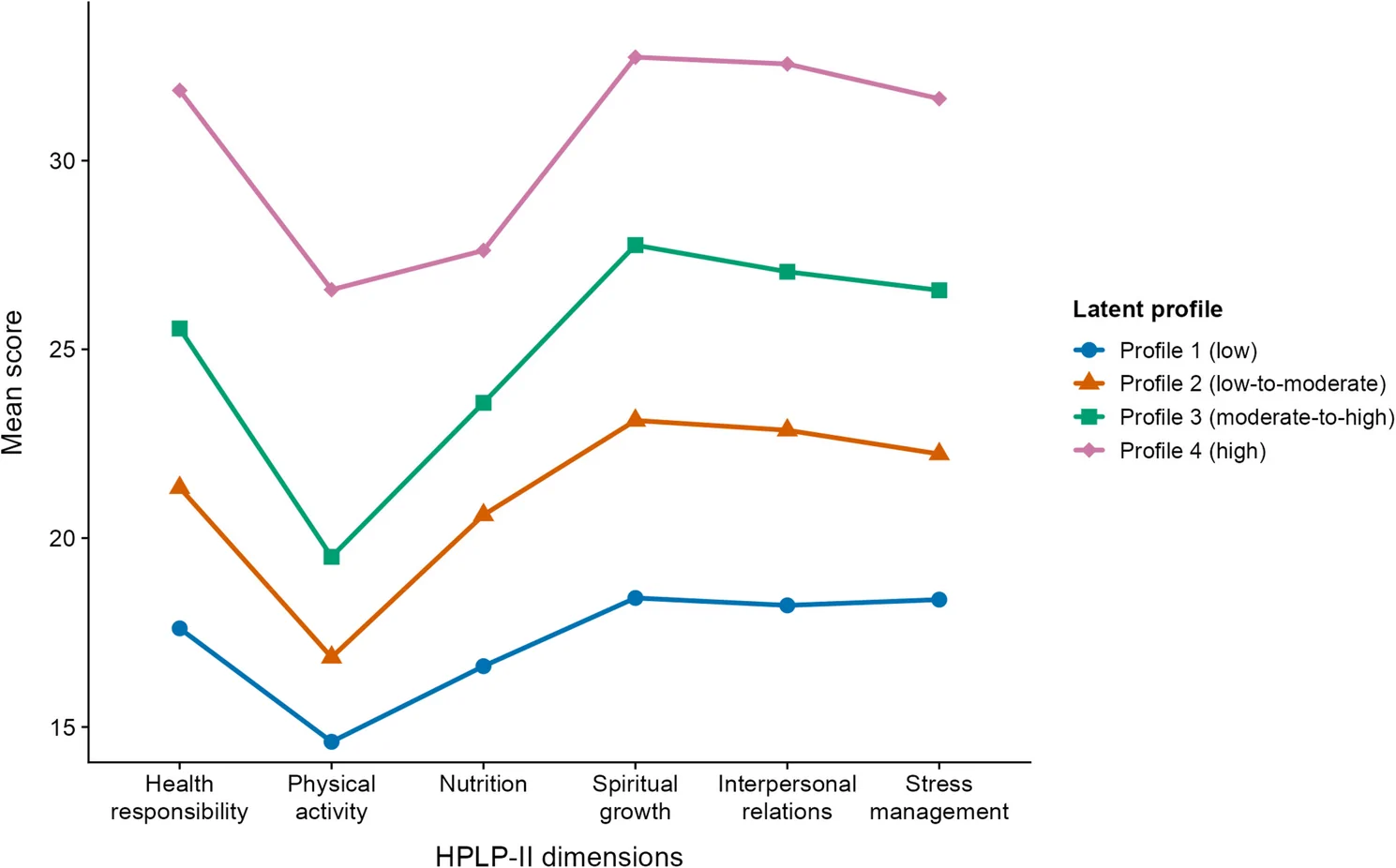
Distribution of scores across the six HPLP-II dimensions among latent health behavior profiles

Figure 1 shows the mean scores across the six HPLP-II dimensions for the four latent profiles. Because latent-profile numbering is arbitrary, the profiles were relabeled and ordered from lower to higher levels of health behavior to facilitate interpretation. Profile 1, designated as the low health-promoting behavior profile (9.7%), showed standardized mean scores ranging from − 1.53 to − 0.76 SD relative to the overall sample mean. Profile 2, designated as the low-to-moderate health-promoting behavior profile (45.5%), showed below-average scores ranging from − 0.49 to − 0.33 SD. Profile 3, designated as the moderate-to-high health-promoting behavior profile (34.3%), showed above-average scores ranging from 0.18 to 0.55 SD. Profile 4, designated as the high health-promoting behavior profile (10.5%), showed markedly elevated scores ranging from 1.48 to 1.77 SD above the overall sample mean.

### Descriptive statistics and comparison between the training and internal validation sets

A total of 475 community residents with clustered cardiovascular risk factors were included in this study, with a mean age of 60.06 ± 15.83 years; 258 participants (54.3%) were male. Among the participants, 214 (45.1%) were smokers, 96 (20.2%) were regular drinkers, 246 (51.8%) had a family history of cardiovascular disease, and 110 (23.2%) had comorbid chronic conditions. The dataset was randomly divided into a training set and an internal validation set at a ratio of 8:2 using stratified sampling, with 381 participants in the training set and 94 in the internal validation set. The distributions of demographic, behavioral, and environmental variables were generally balanced between the two sets, with no statistically significant differences observed in any variables (*P* > 0.05). These results indicated that the baseline characteristics of the training and internal validation sets were comparable, supporting their use for subsequent model development and evaluation. Detailed results are presented in Table 2, and variable assignments are provided in Additional Table S2.

**Table 2:** General characteristics of the training and internal validation sets

Variable	Level	Total	Internal validation set	Training set	*P*
n		475	94	381	
Gender	Female	217 (45.7%)	43 (45.7%)	174 (45.7%)	1.000
	Male	258 (54.3%)	51 (54.3%)	207 (54.3%)	
Occupation	Heavy physical labor	209 (44.0%)	42 (44.7%)	167 (43.8%)	0.974
	Light physical labor	266 (56.0%)	52 (55.3%)	214 (56.2%)	
Marital status	Married	375 (78.9%)	71 (75.5%)	304 (79.8%)	0.368
	Unmarried	45 (9.5%)	10 (10.6%)	35 (9.2%)	
	Divorced	22 (4.6%)	3 (3.2%)	19 (5.0%)	
	Widowed	33 (6.9%)	10 (10.6%)	23 (6.0%)	
Education level	Primary school or below	183 (38.5%)	35 (37.2%)	148 (38.8%)	0.860
	Junior or senior high school	226 (47.6%)	47 (50.0%)	179 (47.0%)	
	Bachelor degree or above	66 (13.9%)	12 (12.8%)	54 (14.2%)	
Monthly per capita household income	≤ 5,000	341 (71.8%)	71 (75.5%)	270 (70.9%)	0.440
	> 5,000	134 (28.2%)	23 (24.5%)	111 (29.1%)	
Family history of disease	No	229 (48.2%)	39 (41.5%)	190 (49.9%)	0.180
	Yes	246 (51.8%)	55 (58.5%)	191 (50.1%)	
Comorbidity of chronic diseases	No	365 (76.8%)	73 (77.7%)	292 (76.6%)	0.942
	Yes	110 (23.2%)	21 (22.3%)	89 (23.4%)	
Smoking status	Non-smoker	261 (54.9%)	49 (52.1%)	212 (55.6%)	0.619
	Smoker	214 (45.1%)	45 (47.9%)	169 (44.4%)	
Alcohol consumption	No or occasional	379 (79.8%)	71 (75.5%)	308 (80.8%)	0.315
	Frequent	96 (20.2%)	23 (24.5%)	73 (19.2%)	
Frequency of health checkups	Frequent	402 (84.6%)	80 (85.1%)	322 (84.5%)	1.000
	Never or occasional	73 (15.4%)	14 (14.9%)	59 (15.5%)	
Residential area	Non-urban	316 (66.5%)	65 (69.1%)	251 (65.9%)	0.632
	Urban	159 (33.5%)	29 (30.9%)	130 (34.1%)	
Type of medical insurance	No medical insurance	30 (6.3%)	5 (5.3%)	25 (6.6%)	0.055
	Resident medical insurance or new rural cooperative medical scheme	400 (84.2%)	86 (91.5%)	314 (82.4%)	
	Employee medical insurance or commercial insurance	45 (9.5%)	3 (3.2%)	42 (11.0%)	
Age	Original value	60.06 ± 15.83	59.62 ± 16.26	60.17 ± 15.74	0.762
Self-efficacy	Original value	24.89 ± 5.03	24.62 ± 4.69	24.95 ± 5.11	0.563
Health behavior capability	Original value	63.28 ± 16.95	62.48 ± 14.86	63.48 ± 17.44	0.608
Social support	Original value	60.36 ± 11.44	61.34 ± 10.73	60.12 ± 11.61	0.354

### Feature selection for health behavior profile classification

LASSO regression and RF were jointly used to screen variables in the training set and identify factors associated with membership in latent health behavior subgroups among community residents with clustered cardiovascular risk factors. Table 3 presents the variable importance scores in the RF model, including MeanDecreaseAccuracy and MeanDecreaseGini, and Table 4 presents the regression coefficients of variables in the LASSO regression model when λ = 0.011. Variables with high MeanDecreaseGini rankings in the RF model and variables with non-zero regression coefficients in the LASSO regression were considered candidate variables for subsequent model development.

**Table 3:** Variable importance rankings from the RF model

Variable	Class 1	Class 2	Class 3	Class 4	MeanDecreaseAccuracy	MeanDecreaseGini
Health behavior capability	40.544	41.742	41.514	42.328	72.607	91.878
Social support	19.271	5.505	23.586	41.704	37.967	55.748
Self-efficacy	7.270	0.609	12.461	10.519	13.022	27.444
Age	2.151	4.626	6.865	8.760	10.752	22.856
Education level	−0.783	1.040	5.366	5.938	5.543	7.188
Marital status	−3.647	−1.815	12.278	4.294	6.171	6.584
Smoking status	0.849	5.353	5.447	7.621	9.680	5.949
Type of medical insurance	−5.416	7.000	13.118	4.272	12.574	4.526
Family history of disease	−4.628	−4.421	9.540	6.789	4.402	4.159
Occupation	−1.036	11.182	6.984	−0.823	11.887	4.158
Monthly per capita household income	−2.783	2.267	5.136	8.720	7.541	3.614
Gender	−3.100	−1.024	3.132	1.118	0.799	3.578
Residential area	−2.369	2.431	2.097	−1.933	1.797	3.325
Comorbidity of chronic diseases	−3.202	3.951	−1.146	0.268	1.638	3.165
Frequency of health checkups	−1.556	2.357	11.967	5.980	9.998	3.066
Alcohol consumption	−3.590	1.963	5.731	1.877	4.849	2.311

**Table 4:** LASSO regression coefficients

Variable	Class 1	Class 2	Class 3	Class 4
(Intercept)	10.082	4.917	−2.990	−12.009
Gender 1	0	0	0	0
Age	0	−0.004	0.017	0
Occupation 1	0	0	−0.630	0
Marital status 2	0	0	0	0
Marital status 3	−0.138	0	0.748	0
Marital status 4	0.156	−0.156	−0.369	0.682
Education level 1	0	0	0	0
Education level 2	0	−0.808	0	0.342
Monthly per capita household income 1	0.055	0	−0.070	0
Family history of disease 1	0	0	−0.446	0
Comorbidity of chronic diseases 1	0	0	0	0
Self-efficacy	−0.018	0.003	0	0
Health behavior capability	−0.109	−0.036	0.036	0.101
Smoking status 1	0	0.218	0	−0.763
Alcohol consumption 1	0	0	−0.051	0
Frequency of health checkups 1	0	0	0	0
Residential area 1	0.250	0.264	−0.250	−0.584
Social support	−0.074	−0.020	0.020	0.073
Type of medical insurance 1	0	−0.005	0.182	0
Type of medical insurance 2	0.345	−0.660	0	0

The coefficients were obtained from the initial variable-selection LASSO model at λ = 0.011

By integrating the results of LASSO regression, RF variable-importance rankings, the SCT framework, and clinical relevance to public health practice, the final predictor set comprised age, occupation, marital status, educational level, monthly per capita household income, family history of disease, self-efficacy, health behavior capability, smoking status, residential area, social support, and type of medical insurance. In contrast, gender, frequency of health checkups, and chronic disease comorbidity were shrunk to zero in the LASSO regression and ranked relatively low in the RF variable-importance analysis. Alcohol consumption had a non-zero LASSO coefficient for only one latent-profile comparison and also showed low importance in the RF analysis. These four variables were therefore excluded from the subsequent classification models to maintain model parsimony and reduce the risk of overfitting. Although their exclusion may reduce the clinical comprehensiveness of the predictor set, it should not be interpreted as evidence that these variables are clinically unimportant, because the present model was designed to classify health-promoting behavior profiles rather than to predict cardiovascular risk or clinical outcomes.

### Performance comparison of machine learning classification models

**Fig. 2:**
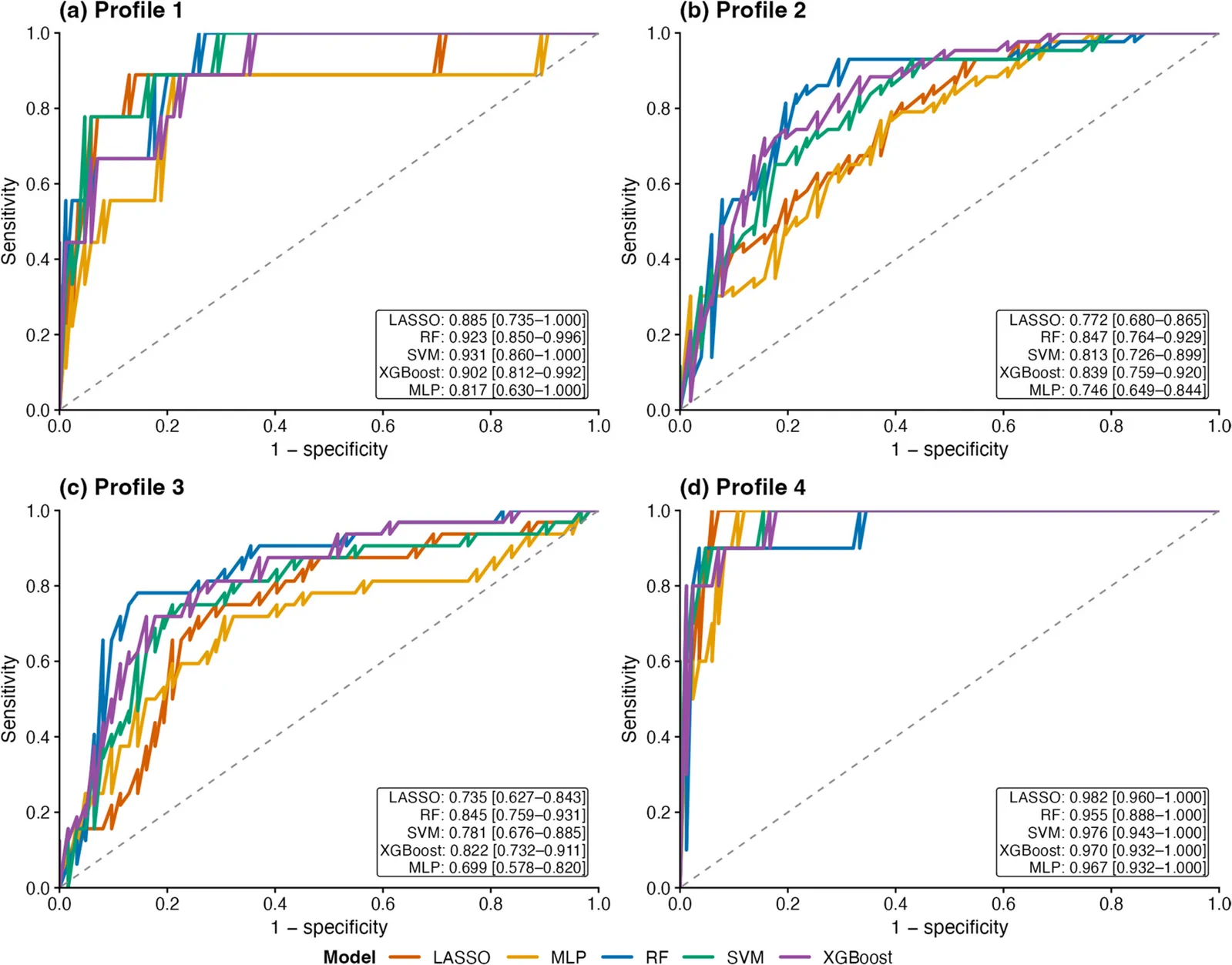
ROC curves for five classification models in the internal validation set. Panels (**a**)-(**d**) show the one-vs-rest ROC curves for Profile 1, Profile 2, Profile 3, and Profile 4, respectively. Values in each panel indicate the AUC and 95% confidence intervals for each model

The 12 variables selected through the final screening process were incorporated into LASSO regression, RF, support vector machine (SVM), XGBoost, and multilayer perceptron (MLP) neural network models to classify LPA-derived health behavior class membership among community residents with clustered cardiovascular risk factors. The performance metrics of the five models in the training and internal validation sets are shown in Table 5. The ROC curves are presented in Fig. 2, and the hyperparameter tuning ranges are provided in Additional Table S3.

**Table 5:** Performance comparison of machine learning classification models in the training and internal validation sets

Model	Dataset	Accuracy	Kappa	Macro Precision	Macro Recall	Macro F1	Weighted Precision	Weighted Recall	Weighted F1	Multiclass AUC
LASSO	training	0.667	0.523	0.628	0.742	0.660	0.699	0.667	0.669	0.907
LASSO	internal validation	0.606	0.422	0.586	0.671	0.617	0.613	0.606	0.604	0.874
RF	training	0.997	0.996	0.994	0.999	0.996	0.997	0.997	0.997	1.000
RF	internal validation	0.777	0.640	0.852	0.671	0.714	0.798	0.777	0.765	0.892
MLP	training	0.727	0.604	0.685	0.788	0.719	0.750	0.727	0.729	0.923
MLP	internal validation	0.585	0.381	0.546	0.608	0.566	0.588	0.585	0.580	0.838
SVM	training	0.793	0.678	0.831	0.784	0.800	0.798	0.793	0.790	0.936
SVM	internal validation	0.691	0.501	0.782	0.594	0.622	0.717	0.691	0.674	0.878
XGBoost	training	0.885	0.828	0.854	0.928	0.884	0.892	0.885	0.885	0.991
XGBoost	internal validation	0.691	0.522	0.662	0.655	0.654	0.691	0.691	0.685	0.895

Macro-averaged metrics assign equal weight to each class, whereas weighted-average metrics weight class-specific estimates according to the number of participants in each class. Multiclass AUC was calculated using the Hand and Till method, which averages pairwise AUC values across all class pairs

*LASSO* Least Absolute Shrinkage and Selection Operator, *RF* Random Forest, *MLP* multilayer perceptron, *SVM* support vector machine, *XGBoost* extreme gradient boosting, *AUC* area under the receiver operating characteristic curve

The results showed differences in the performance of the machine learning classification models between the training and internal validation sets. Although both macro-averaged and weighted-average metrics were reported, macro-averaged metrics were emphasized to assess balanced recognition across all classes, particularly the smaller classes. For the RF model, the training-set accuracy, Kappa, and multiclass AUC were 0.997, 0.996, and 1.000, respectively, whereas its internal-validation-set accuracy and Kappa decreased to 0.777 and 0.640, suggesting potential overfitting. In the internal validation set, RF showed the highest point estimates for accuracy (0.777), Kappa (0.640), macro-averaged precision (0.852), and macro-averaged F1 score (0.714) among the five candidate models, while its macro-averaged recall was 0.671. XGBoost showed a slightly higher multiclass AUC than RF (0.895 vs. 0.892). However, paired stratified bootstrap comparisons showed no statistically significant differences in multiclass AUC between RF and any of the four alternative models; all 95% confidence intervals included zero and all Holm-adjusted P values were greater than 0.05. In particular, the RF-minus-XGBoost AUC difference was − 0.004 (95% CI, − 0.031 to 0.025; Holm-adjusted *P* = 1.000). Considering its favorable descriptive performance across several metrics and its suitability for class-specific interpretation, RF was retained for subsequent exploratory SHAP analysis. This selection should not be interpreted as evidence of statistical superiority over the other candidate models. Nevertheless, the RF model showed low recall for Class 1 (0.333), indicating limited sensitivity for this minority class and the need for further validation. The internal validation set confusion matrix and overall and class-specific performance estimates with bootstrap confidence intervals are provided in Additional Tables S4a–S4c. The complete paired bootstrap comparisons of multiclass AUC are provided in Additional Table S5.

### SHAP-based interpretation of the RF model

**Fig. 3:**
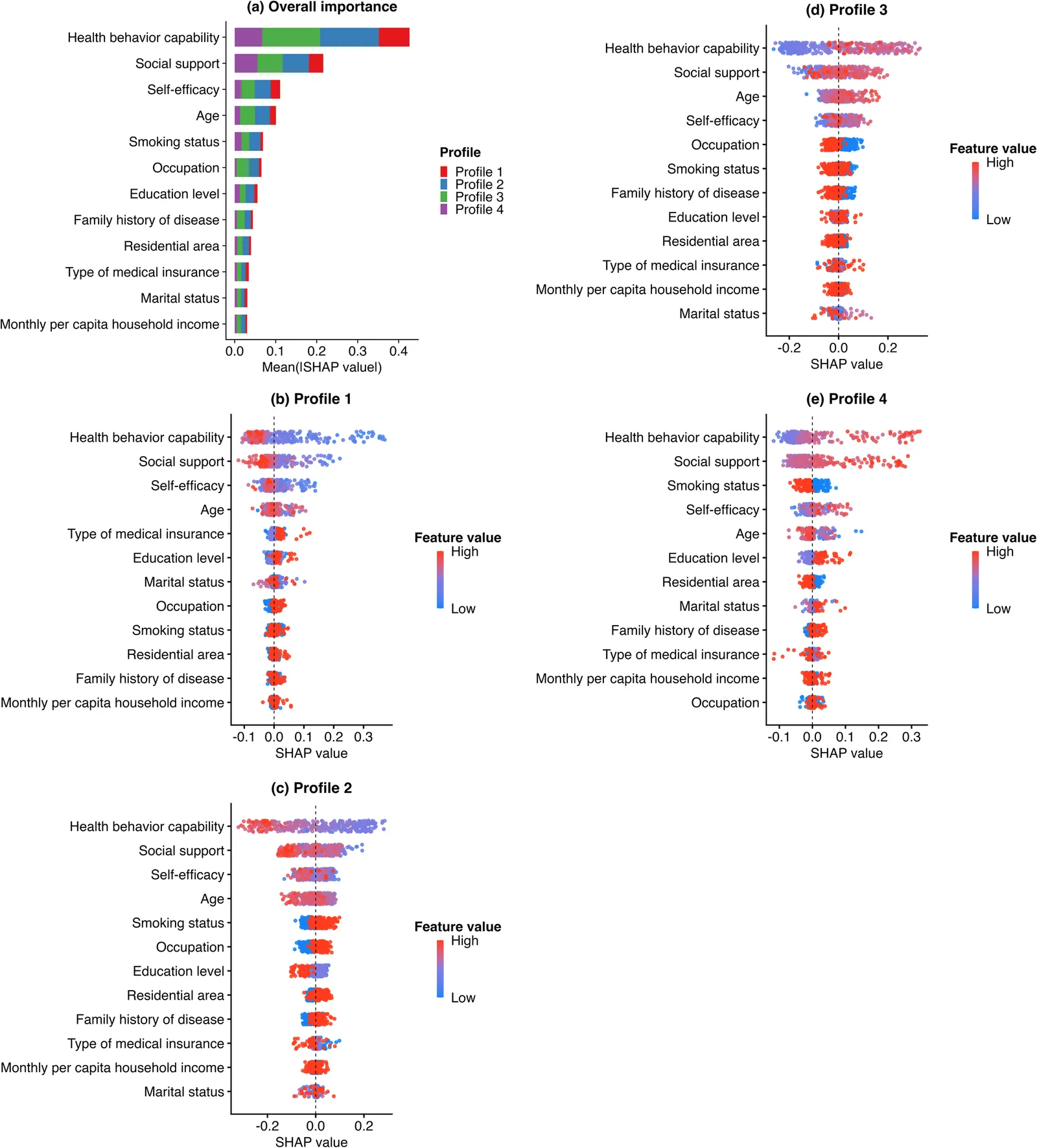
SHAP-based interpretation of the Random Forest model. **a** Overall SHAP feature importance for classifying the four latent health behavior subgroups. **b** SHAP beeswarm plot for Profile 1, the low health-promoting behavior profile. **c** SHAP beeswarm plot for Profile 2, the low-to-moderate health-promoting behavior profile. **d** SHAP beeswarm plot for Profile 3, the moderate-to-high health-promoting behavior profile. **e** SHAP beeswarm plot for Profile 4, the high health-promoting behavior profile. SHAP, Shapley Additive Explanations

SHAP explainability analysis was used to quantify the magnitude and direction of each variable’s contribution to the RF model retained for exploratory interpretation. Figure 3 shows the SHAP-based interpretation of the RF model. Panel (a) presents the overall SHAP feature importance for classifying the four latent health behavior subgroups. Panels (b)–(e) show the SHAP beeswarm plots for Profile 1, Profile 2, Profile 3, and Profile 4, respectively. A SHAP value greater than 0 indicates that the variable contributes positively to classification into that subgroup, whereas a SHAP value less than 0 indicates that the variable contributes negatively to classification into that subgroup. The color represents the relative value of the variable, with red indicating higher values and blue indicating lower values. The SHAP results showed that health behavior capability, social support, self-efficacy, and age had high contributions across the classification of the four subgroups, suggesting that these variables were key contributors to the model’s ability to distinguish different latent health behavior subgroups.

## Discussion

### Latent profiles of health behaviors and stratified management

Based on the HPLP-II, our study identified four latent health behavior subgroups among community residents with clustered cardiovascular risk factors. The health behaviors of these four subgroups showed a clear gradient from low to high across the six dimensions of health responsibility, interpersonal relationships, nutrition, physical activity, spiritual growth, and stress management. This finding suggests that heterogeneity in health behaviors within this population was mainly characterized by differences in the overall level of health-promoting behaviors across multiple dimensions, rather than by variation in a single behavioral dimension. This heterogeneity has practical relevance for community-based prevention services because it suggests that the profiles may provide a preliminary framework for further stratified behavioral assessment. Tegegne et al. [42] found that lifestyle risk behaviors can cluster into distinct subgroups and that long-term outcomes among patients with CVD differ across these subgroups. Identifying these profiles may help generate hypotheses for future stratified intervention studies and provide insights for health behavior management before adverse cardiovascular events occur [43]. In the context of precision medicine, a person-centered approach can help capture heterogeneity in health behaviors within a population and address the limitations of traditional variable-centered methods in identifying individual behavioral differences. This provides a preliminary descriptive framework for future person-centered assessment and intervention research [44]. In community public health work, the findings may help health workers identify differences in health behavior support needs based on subgroup characteristics. In community public health practice, the observed profiles may help identify areas warranting further individualized assessment. Residents with lower observed levels of health behaviors could be assessed for behavioral barriers, health behavior capability, self-efficacy, and social support. In contrast, those with comparatively higher levels could still be assessed for factors affecting the continuation of favorable behaviors. The appropriate form and intensity of support should be determined through individualized assessment and tested in future intervention studies.

The low health-promoting behavior profile had the lowest scores across all six dimensions, suggesting that this group may have an overall insufficient level of health behaviors. This overall low level may indicate limited engagement across multiple health behavior domains, including health responsibility, physical activity, nutrition, stress management, spiritual growth and interpersonal relationship related behaviors. Compared with insufficiency in a single behavioral domain, this pattern suggests a multidimensional insufficiency in health behaviors. Further assessment may be needed to determine whether self-efficacy, health behavior capability, social support, or other barriers contribute to this pattern. In community interventions, this profile may warrant further individualized assessment to determine whether more intensive behavioral support would be appropriate in future stratified community-based prevention services. Future intervention studies could examine whether cardiovascular health education, behavioral skills training, or social support enhancement is effective for residents with this profile.

The low-to-moderate health-promoting behavior profile had higher health behavior scores than the low health-promoting behavior profile but still remained at a relatively low level. This pattern may be consistent with a transitional phase of health behavior change. One possible interpretation is that some residents in this profile may have developed health management awareness but may not yet have consistently translated this awareness into health-promoting practices. From a practice perspective, support for this subgroup may focus on facilitating the transition from health awareness and behavioral capability to behavioral practice. Specifically, health workers could consider helping residents with clustered cardiovascular risk factors strengthen their understanding of cardiovascular health management, acquire practical health behavior skills, and gradually transform existing awareness into actionable and sustained health behaviors.

The moderate-to-high health-promoting behavior profile scored higher than the low health-promoting behavior profile and the low-to-moderate health-promoting behavior profile across the six health behavior dimensions. Still, its scores remained lower than those of the high health-promoting behavior profile. This suggests that this subgroup showed comparatively favorable observed health behavior levels, although further improvement was still needed. Individuals in this subgroup may have established a certain foundation of health behaviors, but the stability and sustainability of these behaviors may remain insufficient. Therefore, future longitudinal studies could evaluate whether monitoring, feedback, or behavioral reinforcement helps maintain or further improve the comparatively favorable behavior levels observed in this subgroup.

The high health-promoting behavior profile had the highest scores across all six dimensions, indicating that this subgroup exhibited a relatively favorable level of health behaviors in this study. However, this finding does not mean that individuals in this subgroup have fully established optimal health behaviors or no longer require behavioral support. These residents may still be exposed to nonmodifiable risk factors, such as age and family history, as well as modifiable risk factors, including hypertension, dyslipidemia, obesity, and smoking. During the dynamic process of chronic disease management, longitudinal research is needed to determine whether favorable behavior levels are maintained when environmental or support conditions change. Therefore, residents in this profile may continue to receive routine assessment and standard community follow-up, while future studies evaluate whether maintenance strategies provide benefit.

Although the observed behavioral profiles may suggest differing levels or types of health behavior support, support needs were not directly measured in this study. Therefore, these implications should be interpreted as cautious inferences derived from differences in health behavior levels rather than as direct evidence of residents’ perceived support needs.

### Factors associated with latent health behavior subgroup membership

Within the framework of SCT and by integrating the results of explainable machine learning, this study identified key factors contributing to the classification of health behavior subgroups. These factors covered personal, behavioral, and environmental factors and may provide a reference for community health workers to conduct stratified assessment and targeted health behavior interventions.

Health behavior capability was the strongest contributor to subgroup classification across all four profiles and a key variable for distinguishing health behavior patterns among community residents with clustered cardiovascular risk factors. According to the National Cancer Institute’s guide to health behavior theory, behavioral capability refers to the knowledge and skills required to perform a specific behavior and is theoretically relevant to the initiation and maintenance of health behavior change [45]. The findings of this study indicate that health behavior capability contributed substantially to distinguishing subgroups with different observed levels of health behaviors. Lower health behavior capability values were more frequently associated with classification into the low and low-to-moderate health promoting behavior profiles. Conversely, higher health behavior capability values were more frequently associated with classification into the moderate to high and high health promoting behavior profiles. This finding is consistent with Bandura’s SCT, which provides a theoretical basis for understanding the role of behavioral capability in the formation and maintenance of health behaviors [46]. Walters et al. [47] found in their study of individuals with CVD that higher levels of health literacy and self-management capability were also associated with more proactive health behaviors.

SHAP analysis indicated that social support and self-efficacy contributed to the classification of individuals into different health behavior subgroups, suggesting that personal beliefs and environmental resources are relevant to distinguishing these subgroups. Within the Health Action Process Approach (HAPA), self-efficacy may support the motivational phase by strengthening individuals’ confidence in initiating health behavior change, whereas health behavior capability and social support may facilitate the translation of behavioral intentions into sustained action during the volitional phase [48, 49]. Self-efficacy reflects an individual’s belief in their ability to initiate and maintain health behavior change, whereas social support represents external resources and environmental support. When individuals receive more health-related informational support, practical assistance, and emotional support from family members, peers, and community health workers, they may be more likely to establish daily health behaviors and thus be classified into the moderate-to-high or high health-promoting behavior profiles. In contrast, insufficient social support may create barriers to initiating health behavior change and maintaining behaviors such as dietary management, physical activity, stress management, and regular health monitoring.

Self-efficacy further helps explain differences in health behaviors within this population. Individuals with higher self-efficacy may be more likely to believe they can manage cardiovascular risk factors and sustain health behavior changes, thereby promoting engagement in multidimensional health behaviors. Conversely, individuals with lower self-efficacy may be more prone to frustration and withdrawal when facing perceived barriers, making it difficult to sustain health behaviors. The partial overlap in self-efficacy between the low-to-moderate and moderate-to-high health-promoting behavior profiles suggests that self-efficacy alone may not be sufficient to distinguish subgroup membership. Actual health behaviors may also depend on volitional-stage factors, including action planning, health behavior capability, and external support, which help translate confidence and intention into sustained action.

Age also contributed to subgroup classification in this study. SHAP analysis indicated that age was particularly important for distinguishing the low-to-moderate health-promoting behavior profile from the moderate-to-high health-promoting behavior profile. The contribution of age in differentiating these two groups among community residents with clustered cardiovascular risk factors may be related to greater perceived disease risk, accumulated illness-related experience, and more frequent engagement with healthcare services. Older individuals may have greater awareness of disease risk and more experience with chronic disease management, which could be associated with greater attention to health-related behaviors. However, advancing age does not necessarily mean that individuals will be classified into the high health-promoting behavior profile. Declining physical function and long-established lifestyle habits associated with aging may also limit improvements in health behaviors such as physical activity and nutrition. These findings suggest that the association between age and health behavior profile membership may not be simply linear. Rather, age may serve as a marker of differences in illness experience, risk perception, healthcare engagement, and physical function, which may interact with health behavior capability, social support, and self-efficacy in differentiating the observed profiles.

SHAP analysis showed that socioeconomic and healthcare resource-related factors, including occupation, educational level, monthly per capita household income, type of medical insurance, and residential area, as well as factors related to family circumstances and risk perception, such as marital status and family history of disease, also provided supplementary information for distinguishing membership in different health behavior subgroups. These factors may capture differences in access to health information, health literacy, healthcare utilization, and perceived disease risk that are theoretically relevant to subgroup differentiation. Smoking status also played a prominent role in distinguishing the high health-promoting behavior profile from the other subgroups. Among community residents with clustered cardiovascular risk factors, non-smoking status may be associated with higher observed levels of health behaviors. From a theoretical perspective, the classification contribution of smoking status may reflect differences in nicotine dependence, risk perception, stress coping, or other unmeasured behavioral and contextual factors.

Guided by the SCT framework, health behavior capability, self-efficacy, and age represented individual-level characteristics; smoking status represented a behavioral factor; and social support represented an environmental factor. The observed classification patterns were not attributable to a single variable but were associated with information across individual, behavioral, and environmental domains. Therefore, these findings suggest that individual, behavioral, and environmental factors may be considered as candidate domains for further assessment when designing and testing future stratified health behavior interventions for community residents with clustered cardiovascular risk factors.

### Machine learning interpretation of factors associated with health behavior profiles

This study combined LPA with explainable machine learning to identify latent health behavior subgroups and their associated factors among community residents with clustered cardiovascular risk factors, thereby providing an exploratory basis for community health workers to conduct stratified assessment and intervention. Compared with traditional regression models, the RF model is capable of capturing complex nonlinear relationships and interactions among variables, making it suitable for classification tasks influenced by multiple factors, including individuals’ intrinsic capabilities, personal beliefs, behavioral habits, and environmental support. In this study, SHAP was used to quantify the magnitude and direction of different variables’ contributions to health behavior subgroup classification. Key contributors, including health behavior capability, social support, self-efficacy, and age, were identified, thereby reducing, to some extent, the limitations associated with the “black-box” nature of machine learning [24]. Although these constructs were assessed using separate instruments and were not used to derive the latent profiles, they are theoretically related to health behaviors; therefore, their SHAP importance should be interpreted as classification-relevant associations rather than independent or causal effects.

### Implications for community-based public health practice

Our findings suggest that community health workers may need to move beyond uniform health education and adopt stratified health behavior management for residents with clustered cardiovascular risk factors. Different subgroups showed distinct observed levels of health behaviors and may therefore warrant consideration of different support priorities, intensities, and forms of cardiovascular prevention services. This provides a preliminary practice-oriented framework for community health workers to consider the organization of health education, behavioral guidance, and follow-up support according to potential subgroup-specific support priorities.

For public health practice, subgroup characteristics help primary care and public health practitioners provide differentiated health education, behavioral guidance, and follow-up support. For the low and low-to-moderate health-promoting behavior profiles, future intervention approaches could focus on health behavior capability and self-efficacy through cardiovascular health education, behavioral skills training, strengthening positive health beliefs, and involving family support networks. For the moderate-to-high and high health-promoting behavior profiles, future support approaches could place greater emphasis on monitoring and reinforcing favorable health behaviors through remote follow-up, periodic self-monitoring, and lower-intensity behavioral support. In addition, smoking status may be considered in health behavior management. Community health workers could consider approaches such as motivational interviewing to assess willingness to quit smoking and nicotine dependence and to provide tailored smoking cessation guidance and follow-up support. Family, peer, and community public health resources may also be mobilized to improve external conditions that support health behavior change.

At the primary care and public health service level, the observed heterogeneity in health behavior profiles raises the possibility that a uniform service approach may not fully address residents’ differing potential support priorities. Subgroup-based information may help community managers and health workers identify groups that may warrant different intensities of behavioral support, plan differentiated follow-up schedules, and align available service resources with residents’ support needs. Because the present study did not measure staffing input, workload, cost, service utilization, or post-intervention patient outcomes, its management implications should be interpreted as a rationale for future implementation and cost-effectiveness studies rather than as direct evidence of resource optimization.

### Limitations

This study has several limitations. First, because of the cross-sectional design, the SHAP results can only reflect the contribution of variables to model classification. They cannot be used to infer causal relationships between individual variables and health behavior subgroup membership. Therefore, the RF model should be interpreted as an exploratory classification model for identifying health behavior subgroup membership rather than as a clinically validated prediction model for risk prediction or decision-making. Second, the overall sample size was relatively limited, and the distribution of participants across latent profiles was imbalanced. Although class weighting was used during model training to improve recognition of minority classes, the low health-promoting behavior profile accounted for only 9.7% of the total sample and included approximately nine participants in the internal validation set. This limited subgroup size likely contributed to the relatively low recall observed for this class and may have resulted in unstable class-specific performance estimates. Larger samples with sufficient representation of smaller latent profiles are needed to improve the reliability of classification, particularly for individuals who may require greater behavioral support. Third, although a stratified splitting strategy was used to create the training and internal validation sets, no independent external validation was conducted. Accordingly, the reported RF performance may be optimistic and should not be interpreted as an unbiased estimate of model performance in new populations. In addition, convenience sampling from eight communities in four cities in Zhejiang Province may have limited the representativeness and generalizability of the findings. Finally, some variables were collected through self-report, which may have introduced recall bias and affected data quality. Taken together, the current RF model remains exploratory and should not yet be used for individual-level clinical or public health decision-making, service allocation, or real-world deployment. External validation in larger, independent, and geographically diverse community cohorts is required. Future studies should expand the sample size, further address class imbalance, incorporate more objective variables, and validate the stability and generalizability of the model in prospective cohort studies and independent external samples.

## Conclusions

Based on a person-centered approach, this study identified four latent health behavior subgroups among community residents with clustered cardiovascular risk factors, indicating marked heterogeneity in health behaviors within this population. Following the exploratory comparison of the candidate models, the RF model was retained for SHAP-based interpretation, which identified health behavior capability, social support, self-efficacy, and age as key contributors to subgroup classification. These findings may help community health workers recognize differences in health behavior across subgroups and generate cautious hypotheses regarding their potential support needs, while informing future research on stratified community-based prevention and chronic disease management.

## Supplementary Material

Additional file 1: Additional Table S1. Average posterior probabilities for the four profile latent profile analysis model.

Additional file 2: Additional Table S2. Variable coding.

Additional file 3: Additional Table S3. Hyperparameter tuning ranges and final parameters.

Additional file 4: Additional Tables S4a–S4c. RF internal validation set confusion matrix and overall and class specific performance estimates with 95% bootstrap confidence intervals.

Additional file 5: Additional Table S5. Paired bootstrap comparisons of multiclass AUC between the RF model and the alternative models.

Additional file 6: Additional Table S6. Key methodological details for reproducibility.

## Data Availability

The datasets generated and analyzed during the current study are not publicly available due to participant privacy and ethical restrictions, but are available from the corresponding author on reasonable request.

## References

[1] Bays HE, Taub PR, Epstein E, Michos ED, Ferraro RA, Bailey AL, et al. Ten things to know about ten cardiovascular disease risk factors. Am J Prev Cardiol. 2021;5:100149. 10.1016/j.ajpc.2021.100149. PMC8315386 34327491

[2] World Health Organization. Cardiovascular diseases (CVDs). 2025. https://www.who.int/news-room/fact-sheets/detail/cardiovascular-diseases-%28cvds%29. Accessed 2026.

[3] Mensah GA, Fuster V, Murray CJL, Roth GA. Global Burden of Cardiovascular Diseases and Risks Collaborators. Global burden of cardiovascular diseases and risks, 1990–2022. J Am Coll Cardiol. 2023;82:2350–473. 10.1016/j.jacc.2023.11.007. PMC7615984 38092509

[4] Global Burden of Cardiovascular Diseases and Risks. 2023 Collaborators. Global, regional, and national burden of cardiovascular diseases and risk factors in 204 countries and territories, 1990–2023. J Am Coll Cardiol. 2025;86:2167–243. 10.1016/j.jacc.2025.08.015. 40990886

[5] Davies MJ, Drexel H, Jornayvaz FR, Pataky Z, Seferović PM, Wanner C. Cardiovascular outcomes trials: A paradigm shift in the current management of type 2 diabetes. Cardiovasc Diabetol. 2022;21:144. 10.1186/s12933-022-01575-9. PMC9351217 35927730

[6] Holthuis EI, Visseren FLJ, Bots ML, Peters SAE. Risk factor clusters and cardiovascular disease in high-risk patients: the UCC-SMART study. Glob Heart. 2021;16:85. 10.5334/gh.897. PMC8698229 35141126

[7] World Health Organization. HEARTS: technical package for cardiovascular disease management in primary health care: risk-based CVD management. Geneva: World Health Organization; 2020.

[8] Kasl SV, Cobb S. Health behavior, illness behavior, and sick role behavior. I. Health and illness behavior. Arch Environ Health. 1966;12:246–66. 10.1080/00039896.1966.10664365. 5322534

[9] Arnett DK, Blumenthal RS, Albert MA, Buroker AB, Goldberger ZD, Hahn EJ, et al. 2019 ACC/AHA guideline on the primary prevention of cardiovascular disease: a report of the American College of Cardiology/American Heart Association Task Force on Clinical Practice Guidelines. Circulation. 2019;140:e596–646. 10.1161/CIR.0000000000000678. PMC7734661 30879355

[10] Deng W, Wang D, Wan Y, Lai S, Ding Y, Wang X. Prediction models for major adverse cardiovascular events after percutaneous coronary intervention: A systematic review. Front Cardiovasc Med. 2024;10:1287434. 10.3389/fcvm.2023.1287434. PMC10800829 38259313

[11] Zhai Y, Shang H, Li Y, Zhang N, Zhang J, Wu S. A systematic review of risk factors for major adverse cardiovascular events in patients with coronary heart disease who underwent percutaneous coronary intervention. Front Physiol. 2025;16:1514585. 10.3389/fphys.2025.1514585. PMC12014560 40271216

[12] Nguyen ATH, Saeed A, Bambs CE, Swanson J, Emechebe N, Mansuri F, et al. Usefulness of the American Heart Association’s ideal cardiovascular health measure to predict long-term major adverse cardiovascular events (from the Heart SCORE Study). Am J Cardiol. 2021;138:20–5. 10.1016/j.amjcard.2020.10.019. 33065086

[13] Wei L. Factors influencing major adverse cardiovascular events after PCI in patients with acute myocardial infarction [in Chinese]. Chin J Cardiovasc Rehabilitation Med. 2018;27:633–7.

[14] Duan Y, An Y, Wang J. Current status and influencing factors of negative emotions, quality of life, and adverse cardiovascular events in elderly patients with chronic heart failure [in Chinese]. Chin J Multiple Organ Dis Elder. 2023;22:852–6.

[15] Howard MC, Hoffman ME. Variable-centered, person-centered, and person-specific approaches: where theory meets the method. Organ Res Methods. 2018;21:846–76. 10.1177/1094428117744021.

[16] Muthén B, Muthén LK. Integrating person-centered and variable-centered analyses: growth mixture modeling with latent trajectory classes. Alcohol Clin Exp Res. 2000;24:882–91. 10.1111/j.1530-0277.2000.tb02070.x. 10888079

[17] Huang Z, Ho JS, Yap QV, Chan YH, Tan SY, Ya NKS, et al. Patterns of motivators and barriers to heart health behaviors among adults with behavior-modifiable cardiovascular risk factors: a population-based survey in Singapore. PLoS ONE. 2022;17:e0262752. 10.1371/journal.pone.0262752. PMC8775312 35051229

[18] Park H-C, Oh J. Factors affecting health-promoting behaviors in patients with cardiovascular disease. Healthc (Basel). 2021;9:60. 10.3390/healthcare9010060. PMC7827905 33435583

[19] Dinh TTH, Bonner A. Exploring the relationships between health literacy, social support, self-efficacy and self-management in adults with multiple chronic diseases. BMC Health Serv Res. 2023;23:923. 10.1186/s12913-023-09907-5. PMC10466814 37649013

[20] Alageel S, Gulliford MC. Health checks and cardiovascular risk factor values over six years’ follow-up: matched cohort study using electronic health records in England. PLoS Med. 2019;16:e1002863. 10.1371/journal.pmed.1002863. PMC6667114 31361740

[21] Bandura A. Social cognitive theory: An agentic perspective. Annu Rev Psychol. 2001;52:1–26. 10.1146/annurev.psych.52.1.1. 11148297

[22] Rajula HSR, Verlato G, Manchia M, Antonucci N, Fanos V. Comparison of conventional statistical methods with machine learning in medicine: Diagnosis, drug development, and treatment. Med (Kaunas). 2020;56:455. 10.3390/medicina56090455. PMC7560135 32911665

[23] Sidey-Gibbons JAM, Sidey-Gibbons CJ. Machine learning in medicine: A practical introduction. BMC Med Res Methodol. 2019;19:64. 10.1186/s12874-019-0681-4. PMC6425557 30890124

[24] Rudin C. Stop explaining black box machine learning models for high stakes decisions and use interpretable models instead. Nat Mach Intell. 2019;1:206–15. 10.1038/s42256-019-0048-x. PMC9122117 35603010

[25] Chen H, Covert IC, Lundberg SM, Lee S-I. Algorithms to estimate Shapley value feature attributions. Nat Mach Intell. 2023;5:590–601. 10.1038/s42256-023-00657-x.

[26] Lundberg SM, Erion G, Chen H, DeGrave A, Prutkin JM, Nair B, et al. From local explanations to global understanding with explainable AI for trees. Nat Mach Intell. 2020;2:56–67. 10.1038/s42256-019-0138-9. PMC7326367 32607472

[27] Liu C, Lian N, Sznajder KK, Li C, Zou C, Yang X. Characterizing sleep disturbance subgroups and identifying associated factors in traditional Chinese medicine nurses: a latent profile analysis and explainable machine learning approach. J Nurs Manag. 2026;2026:1269507. 10.1155/jonm/1269507. PMC12931154 41743461

[28] Lwanga SK, Lemeshow S. Sample size determination in health studies: a practical manual. Geneva: World Health Organization; 1991.

[29] Healthy China Action Promotion Committee. Healthy China Action (2019–2030) [in Chinese]. 2019. https://www.nhc.gov.cn/guihuaxxs/c100133/201907/2a6ed52f1c264203b5351bdbbadd2da8.shtml. Accessed 2026.

[30] Chinese Society of Cardiology of Chinese Medical Association, Cardiovascular Disease Prevention and Rehabilitation Committee of Chinese Association of Rehabilitation Medicine, Cardiovascular Disease Committee of Chinese Association of Gerontology and Geriatrics, Thrombosis Prevention and Treatment Committee of Chinese Medical Doctor Association. Chinese guideline on the primary prevention of cardiovascular diseases [in Chinese]. Zhonghua xin xue guan bing za zhi. 2020;48:1000–38. 10.3760/cma.j.cn112148-20201009-00796. 33355747

[31] Zhang Y, Ren L, Ren M, Yang H, Li K, Cong H, et al. Correlation Between the Triglyceride–Glucose Index and High Risk of Cardiovascular Disease: A Cohort Study of 102,061 Subjects from Tianjin, China. Risk Manag Healthc Policy. 2021;14:2803–10. 10.2147/RMHP.S316484. PMC8260047 34239336

[32] Zhou C, Zheng W, Tan F, Lai S, Yuan Q. Influence of health promoting lifestyle on health management intentions and behaviors among Chinese residents under the integrated healthcare system. PLoS ONE. 2022;17:e0263004. 10.1371/journal.pone.0263004. PMC8789132 35077472

[33] Walker SN, Sechrist KR, Pender NJ. The Health-Promoting Lifestyle Profile: Development and Psychometric Characteristics. Nurs Res. 1987;36:76–81. 3644262

[34] Xiao L, Wang P, Fang Q, Zhao Q. Health-promoting lifestyle in patients after percutaneous coronary intervention. Korean Circ J. 2018;48:507–15. 10.4070/kcj.2017.0312. PMC5986750 29856145

[35] Schwarzer R, Jerusalem M. Generalized Self-Efficacy Scale. In: Weinman J, Wright S, Johnston M, editors. Measures in Health Psychology: A User’s Portfolio. Causal and Control Beliefs. Windsor, UK: NFER-NELSON; 1995. pp. 35–7.

[36] Zhang JX, Schwarzer R. Measuring optimistic self-beliefs: A Chinese adaptation of the General Self-Efficacy Scale. Psychologia. 1995;38:174–81.

[37] Wang CK, Hu ZF, Liu Y. Evidences for reliability and validity of the Chinese version of General Self-Efficacy Scale [in Chinese]. Chin J Appl Psychol. 2001;7(1):37–40. 10.3785/1006-6020.2001.01.0037.

[38] Becker H, Stuifbergen A, Oh HS, Hall S. Self-rated abilities for health practices: A health self-efficacy measure. Health Values. 1993;17:42–50.

[39] Huang YH, Chiou CJ. Predictors contributing to health-promoting lifestyles among college students in Kaohsiung area [in Chinese]. Chin J Public Health. 1997;16(1):24–36. 10.6288/CJPH1997-16-01-03.

[40] Zimet GD, Dahlem NW, Zimet SG, Farley GK. The Multidimensional Scale of Perceived Social Support. J Pers Assess. 1988;52:30–41. 10.1207/s15327752jpa5201_2.

[41] Jiang QJ. Perceived social support scale [in Chinese]. Chin J Behav Med Sci. 2001;10:41–3.

[42] Tegegne TK, Islam SMS, Maddison R. Effects of lifestyle risk behavior clustering on cardiovascular disease among UK adults: latent class analysis with distal outcomes. Sci Rep. 2022;12:17349. 10.1038/s41598-022-22469-6. PMC9576714 36253519

[43] Luo J, Dinh P, Hendryx M, Li W, Robinson J, Margolis K. Risk patterns and mortality in postmenopausal women using latent class analysis. Am J Prev Med. 2021;61:e225–33. 10.1016/j.amepre.2021.04.025. PMC8542102 34686303

[44] Yang Q, Zhao A, Lee C, Wang X, Vorderstrasse A, Wolever RQ. Latent profile/class analysis identifying differentiated intervention effects. Nurs Res. 2022;71:394–403. 10.1097/NNR.0000000000000597. 35417442

[45] National Cancer Institute. Theory at a glance: a guide for health promotion practice. 2nd edition. Bethesda, MD: National Cancer Institute, National Institutes of Health, U.S. Department of Health and Human Services; 2005. NIH Publication No. 05-3896. Accessed 30 July 2026. https://cancercontrol.cancer.gov/sites/default/files/2020-06/theory.pdf

[46] Bandura A. Health promotion by social cognitive means. Health Educ Behav. 2004;31:143–64. 10.1177/1090198104263660. 15090118

[47] Walters R, Leslie SJ, Polson R, Cusack T, Gorely T. Establishing the efficacy of interventions to improve health literacy and health behaviours: a systematic review. BMC Public Health. 2020;20:1040. 10.1186/s12889-020-08991-0. PMC7329558 32605608

[48] Scholz U, Schüz B, Ziegelmann JP, Lippke S, Schwarzer R. Beyond behavioural intentions: planning mediates between intentions and physical activity. Br J Health Psychol. 2008;13:479–94. 10.1348/135910707X216062. 17553212

[49] Schwarzer R, Lippke S, Luszczynska A. Mechanisms of health behavior change in persons with chronic illness or disability: the health action process approach (HAPA). Rehabil Psychol. 2011;56:161–70. 10.1037/a0024509. 21767036

