# Supplementary material for: Health behavior profiles and potential support needs among community residents with clustered cardiovascular risk factors: a latent profile and explainable machine learning analysis for community-based prevention
Source: BMC Public Health. 2026 Jul 31;26:3094. doi: 10.1186/s12889-026-28868-y (PMC13715248; doi:10.1186/s12889-026-28868-y)
Supplement: Supplementary file 1 [file 12889_2026_28868_MOESM6_ESM.docx]

Additional Table S6. Key methodological details for reproducibility

**Latent profile analysis, machine-learning comparison, and SHAP implementation**

| **Analytical component** | **Implementation details** |
| --- | --- |
| LPA implementation | LPA was conducted in R 4.5.3 using tidyLPA 2.0.2 with mclust 6.1.2 as the estimation engine. One- to six-profile solutions were estimated using model 1 (EEI) based on six standardized HPLP-II dimension scores. |
| Random starts, convergence, and assignment | No user-defined numbers of random starts, maximum iterations, or convergence thresholds were specified; package-default settings were used. LPA estimation used seed 1234. Participants were assigned to the profile with the highest posterior probability. |
| Data partition and cross-validation | An 8:2 stratified training–internal-validation split was generated using caret::createDataPartition with seed 12345. Hyperparameter tuning was conducted within the training set using 10-fold cross-validation with seed 123. |
| Predictor coding and preprocessing | Categorical predictors were represented as factors, with treatment contrasts used where model matrices were required. Continuous-variable preprocessing was model-specific. Variable coding is provided in Additional Table S2. |
| Final model settings | The final RF model used 1,000 trees, mtry = 4, and inverse-frequency class weights. Complete tuning ranges and final hyperparameters for all five models are provided in Additional Table S3. |
| SHAP implementation | SHAP values were approximated using fastshap::explain() version 0.1.1 with the training predictors as background data, nsim = 100, adjust = TRUE, class-probability predictions, and seed 2024. Feature importance was summarized using mean absolute SHAP values. |
